# Improved noise reduction for nonlinear PMDC motor dead zone and friction model using variants of extended Kalman filter with practical validation

**DOI:** 10.1371/journal.pone.0336377

**Published:** 2025-11-12

**Authors:** Shafiq Haider, Sadaqat Ali, Muhammad Saqlain, Akhtar Rasool, Abdulkerim Sherefa

**Affiliations:** 1 Department of Electronics Engineering, University of Engineering and Technology, Taxila, Pakistan; 2 Department of Electrical Engineering, University of Botswana, Gaborone, Botswana; 3 Department of Electrical and Computer Engineering, Wolkite University, Wolkite, Ethiopia; Donghua University, CHINA

## Abstract

An improved framework for measurement noise reduction of nonlinear PMDC motor using variants of extended Kalman filter (EKF) is presented in this paper. Simulatory as well as experimental testing and validation of presented developments has also been performed. The nonlinearities like hard dead zone and friction have been incorporated in the PMDC motor model. Position as well as velocity measurement scenarios have been considered. Firstly, the noise corrupted measurement is invoked in standard EKF that perform prediction and correction to generate the best possible reduced noise estimate of the true measurement. One drawback standard EKF is that it ignores the effect of noise in the physical system and setting process and measurement covariance values in a vague manner that cause inaccurate estimates. In order to remedy this problem, an adaptive variant of EKF is introduced that utilizes the weighting coefficients and forgetting factor in order to set covariance parameters accurately and hence measurement noise reduction and estimation results get relatively accurate. The propositions are tested for angular position and velocity applications through simulation as well as practical experimentation. The results indicate that the adaptive AEKF provides quantitative improvements over the traditional EKF significantly by adaptively adjusting noise covariance matrices. In addition, it is observed that AEKF produce smaller root mean square errors in state estimation, enhance convergence speed, and demonstrate higher tolerance to unforeseen disturbances. These improvements make AEKF particularly valuable in applications such as PMDC machines, navigation systems, robotics, and sensor fusion, where precise and reliable state estimation is critical.

## 1. Introduction

In real-world applications, modeling, controller design, noise cancellation, and disturbance rejection are crucial steps in electromechanical systems. A nonlinear approach to modeling and identification is required for controlling systems that must function under variable conditions or with a high degree of precision. The majority of electromechanical systems found in industrial settings consist of masses that move while being affected by forces that are dependent on both location and velocity. In some operating regions, these forces behave in a nonlinear manner. When the direction of rotation changes in a multi-mass rotating system, nonlinearities such as dead zone and Coulomb friction have a major impact on the system’s performance. Real-time experiments and nonlinear modeling of a DC motor rotating in two directions are presented in the study in [1]. For the goal of identification, the system’s linear and nonlinear models are obtained. The important nonlinearities, such as dead zone and Coulomb friction, are then examined and incorporated into the nonlinear model. The nonlinear system model is identified using the Hammerstein nonlinear system technique. Recursive least square is the method used to identify the linear and nonlinear system models online. The benefits of the nonlinear identification strategy are demonstrated through the graphical and numerical presentation of the real-time experiment results. Similar research is discussed in [2], where real-time data is used to identify and validate the non-linear model of a permanent magnet DC motor. The non-linearities of the so-called dead zone and Coulomb plus viscous friction, which have a substantial impact on electric motors as position actuators, are included in the model. The “hard dead zone” modeling approach, which is thought to provide an accurate representation of the phenomenon, is used to simulate the dead zone. The nonlinear model that is produced is validated and compared with the Maxon DC motor responses of the Quanser DC motor control trainer system using Matlab®/Simulink.

Regarding nonlinear modeling of PMDC motor, [3] presented a technique that consider nonlinear analytic model of a PMDC motors with friction and cogging. An automated identification mechanism is inferred for this detailed model in addition to the theoretical modeling. The electromechanical and electromagnetic effects of the direct current machine, such as motor torque or voltage induction, as well as other nonlinear phenomena, are included in the final model. Cogging torque, eddy current, hysteresis losses, and tribological features are some of these nonlinearities. The torque curve exhibits a periodic oscillation that is attributed to a fluctuation in the magnetic flux density, causing the cogging torque. Furthermore, hysteresis losses and eddy currents caused by the armature’s magnetic field commuting are also included in the motor model. The elastoplastic friction model is used to model the tribological features of all friction regimes. Both the velocity-dependent friction behavior of the plastic friction domain and the linear spring damper behavior of the elastic friction domain can be represented by this model. Through particular experiments that make reference to their physical equivalents, the parameters are independently determined.

Nonlinear Kalman filtering techniques are thought to be appropriate solutions to estimate problems where uncertainty and noise exist in real-world scenarios containing noise and disturbances. Cubature Kalman filtering (CKF), Unscented (UKF), and Extended (EKF) are the three main and fundamental algorithms. The use of these techniques for rotor angular position estimate is discussed in [4], with a focus on low speed states. An experimental setup is used to compare performance measures. The setup uses a standard 3-phase low voltage BLDC motor in order to reduce the possibility of system noise lowering the quality of the Back-EMF signal when it is operating in low speed mode. Measurable improvements in performance and outcomes over EKF are demonstrated by UKF [5–7] and CKF approaches. Unscented and Cubature types perform better in terms of accuracy and convergence, according to estimated model states diagrams. Furthermore, the control algorithm for the motor in non-linear BLDC motor systems has been designed using the Extended Kalman Filter (EKF) in [8]. The stator line voltage and current measurements alone are used in the proposed study to estimate the motor state variables using an Extended Kalman Filter. The simulation findings have been confirmed, and the estimated rotor speed has been used for the BLDC motor’s closed loop speed control.

The rotor position and speed of BLDC motors are estimated by Ensemble Kalman Filter in [9] using only the stator line voltage and current measurement in order to minimize sensor costs and related measurement noise. A recursive filter appropriate for non-linear systems is the Ensemble Kalman Filter. The BLDC motor’s closed loop speed control and motor driving have been implemented using the estimated rotor speed and position rotor. Real-time simulation is used to show how well the suggested technique performs. A new neuro-adaptive control strategy is presented in [10] for tracking angular velocity in a permanent magnet DC motor system controlled by a DC–DC buck converter. To assure nominal tracking performance and counteract the unknown non-linear time-varying load torque, the controller leverages on the concept of back stepping and consists of a fast single hidden layer Hermite neural network (HNN) module with on-board (adaptive) learning. In addition to being computationally efficient, the HNN’s straightforward design shows promise for speed and accuracy in estimating dynamic fluctuations in the unknown load torque. Under parametric and non-parametric uncertainty, the suggested approach in [10] ensures a quick return of nominal angular velocity tracking. The transient reaction behavior is measured using performance indicators like peak undershoot/overshoot and settling time. The findings unequivocally validate theoretical claims and show improved dynamic speed tracking across a broad working range, supporting the applicability of the suggested approach for quick industrial applications.

A well-developed study using the Extended Kalman Filter (EKF) as a nonlinear speed and position observer in PMSM drivers was published in [11]. The PMSM drive system’s poor low speed performance, which produced the best estimation performance in the high speed range (≫5 Hz), was a major issue, though. In the work [11], a novel approach for low speed EKF sensorless control of permanent magnet synchronous motor (PMSM) drives was proposed: adjustable DC bus voltage. Also given were the experiment’s results, which showed that modifying the DC Bus voltage in accordance with the system reference speed improved performance at low speeds (≫2 Hz). A novel finite-time nonlinear extended state observer (NLESO) was proposed and used in active disturbance rejection control (ADRC) to stabilize a nonlinear system against system uncertainties and discontinuous disturbances using output feedback based control [12] with regard to noise and disturbance rejection in PMDC motors. The first step involved combining all of the system’s uncertainties, disturbances, and other undesirable nonlinearities into a single term known as the generalized disturbance. As a result, the NLESO assesses the generalized disturbance and uses an online approach to cancel it from the input channel. The suggested nonlinear ESO (NLESO) greatly reduces a peaking phenomenon that was present in linear ESO (LESO) by using a saturation-like nonlinear function. Finite-time Lyapunov theory is used to study the stability analysis of the NLEO, and comparisons with simulations on permanent magnet DC (PMDC) motors are shown to validate the efficacy of the suggested observer with respect to LESO. The real-world industrial issue of seat positioning motors utilized in higher category cars was the focus of the research in [13]. Initially, the manufacturer’s end-control noise evaluation method is examined and contrasted with lab results. In the second section, a sophisticated model of vibration and noise generation is established, potential pathways for vibration and noise transfer are examined, and the dominant channel is determined. The last section describes some changes made to sets of motors and how they reduced noise. The most successful adjustments were applied cumulatively, resulting in an average noise level reduction of 6–8 dB(A). Additional research is available in [14–19,20].

One thing to keep in mind when evaluating the limitations of EKF is that, in contrast to its linear counterpart, the extended Kalman filter is not, in general, an optimal estimator (it is, however, optimal in cases where the state transition model and the measurement are both linear, in which case the extended Kalman filter is the same as the regular one). Furthermore, because of its linearization, the filter may rapidly diverge if the initial estimate of the state is off or if the process is poorly described. An additional issue with the extended Kalman filter is to its tendency for the estimated covariance matrix to underestimate the true covariance matrix, hence increasing the danger of statistical inconsistency in the absence of “stabilizing noise” [21]. Notably, even for extremely basic one-dimensional systems like the cubic sensor—where the ideal filter may be bimodal [13] and cannot be adequately represented by a single mean and variance estimator, possessing a rich structure—the extended Kalman filter may yield subpar results. The quadratic sensor may also exhibit poor performance in this regard. In order to cater for the technical constraints of EKF for accurate position estimation of nonlinear PMDC motor model, this paper consider the implementation of adaptive EKF with weighting function and forgetting factor that exhibit low error covariance characteristics as compared to traditional EKF version. This study is very essential to correctly judge the angular position and velocity of the PMDC motor in noisy environments.

Adaptive Extended Kalman Filter (AEKF) used in current paper demonstrate better performance for estimation compared to the traditional EKF, mainly through dynamically adapting noise covariance matrices to enhance estimation performance. In contrast to the traditional EKF, which depends on pre-specified and frequently erroneous noise parameters, AEKF adapts these values constantly based on real-time observations, resulting in accurate and stable state estimations. This flexibility improves the filter’s performance against system uncertainties, non-stationary noise, and modeling errors, making it more resilient to real-world implementation. AEKF also enhances convergence rate and decreases estimation errors, especially in extremely dynamic or volatile environments. All these advantages make AEKF superior for use in applications like autonomous navigation, robotics, and sensor fusion, where accurate and trustworthy state tracking is critical. Therefore, accurate estimates are produced by employing this filter to adaptively rectify the statistical features of measurement noise through the use of a forgetting factor. The structure of the paper is as follows. The PMDC motor modeling and controller design challenge is formulated and described in Section 2, and the framework for noise reduction and output estimation is presented in Section 3. The simulation and real-world results for PMDC motor position and velocity control are shown in Section 4, after which the paper is finished.

## 2. Modeling and control design for PMDC motor

The presence of process and measurement noise in physical systems pose a challenge to extract true measurement values when sensor is deployed to measure a certain physical parameter in practical applications. In this regard, in this section, improved technique for true value approximation are presented. The section starts with development of nonlinear mathematical model of the PMDC motor as discussed next.

### 2.1. Nonlinear model of PMDC motor

**Fig 1:**
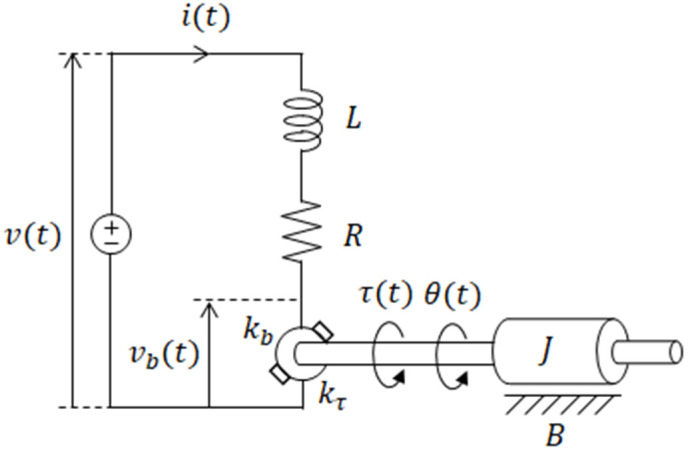
Electromechanical model of PMDC motor.

As PMDC motor is an electromechanical system, its modeling is done with two perspectives, i.e., modeling of electric circuit and mechanical forces and torques. The generic electromechanical model of PMDC motor is shown in Fig 1.

As depicted in Fig 1, as initial step, the PMDC motor can be modeled using two linear equations for the electrical and mechanical subsystems.


$$v(t)=Ri(t)+L\frac{di(t)}{dt}+E_{a}$$ (1)

$$T_{m}=K_{m}i(t)+J\frac{d^{\text{2}}\theta (t)}{dt^{\text{2}}}+b\frac{d\theta (t)}{dt}$$ (2)

The electrical subsystem is represented by equation (1) above, where L and R stand for the resistance and inductance of the armature winding, respectively, and v(t) for the applied armature voltage, i(t) for the armature current, and $E_{a}$ for the counter electromotive force. The mechanical subsystem is represented by the second expression (2), where the rotor’s angular position (θ), the friction coefficient (B), the motor constant ($K_{m}$), the magnetic torque ($T_{m}$), and the motor’s rotor equivalent moment of inertia (J). It is assumed in this model that the counter electromotive force in the motor is very small. Since the counter electromotive force is negligible at low speeds, the electrical subsystem’s transfer function $G_{e}(s)$ ignores it, leading to:


$$G_{e}(s)=\frac{I(s)}{V(s)}=\frac{\frac{\text{1}}{R}}{\left(\frac{L}{R}s+\text{1}\right)}=\frac{K_{e}}{\left(\tau_{e}s+\text{1}\right)}$$ (3)

where 1/R gives the electrical subsystem’s steady state gain $K_{e}$ and $\tau_{e}=\;L/R$ gives the time constant. If we take the rotor velocity to be ω(t= θ, then expression (4) is the transfer function for the mechanical subsystem.


$$G_{m}(s)=\frac{\omega (s)}{T_{m}(s)}=\frac{\frac{\text{1}}{b}}{\left(\frac{J}{b}s+\text{1}\right)}\;;\;T_{m}(s)=k_{m}\text{I}(s)$$ (4)

Thus, the following is the transfer function that links the rotor’s velocity, ω(t), to the input voltage, v(t):


$$\frac{\omega (s)}{\text{V}(s)}=\frac{\frac{k_{m}}{Rb}}{\left(\frac{J}{b}s+\text{1})(\frac{L}{R}s+\text{1}\right)}$$ (5)

Pole dominance allows for the simplification of transfer function (5) because the electrical mode is quicker than the mechanical mode [10,17]. As a result, the PMDC motor transfer function becomes:


$$\frac{\omega (s)}{\text{V}(s)}=\frac{\frac{k_{m}}{R_{b}}}{\left(\frac{J}{b}s+\text{1}\right)}=\frac{K_{mot}}{\tau_{mot}s+\text{1}}$$ (6)

where $\tau_{mot}\;=\;J/b$ is the time constant and $k_{m}/R_{b}$ is the steady state gain of the PMDC motor $K_{mot}$ It is evident from (3) and (4) that the analysis of step responses can be used to determine the transfer functions of mechanical and electrical subsystems.

As the consideration of nonlinear anomalies present in physical system into mathematical model helps to make system analysis and design process more optimal and accurate, therefore PMDC model with dead zone and frictional anomalies has been built in present work to obtain accurate results as discussed in next subsections.

#### 2.1.1. Modeling of dead zone in PMDC motor characteristic curve.

**Fig 2:**
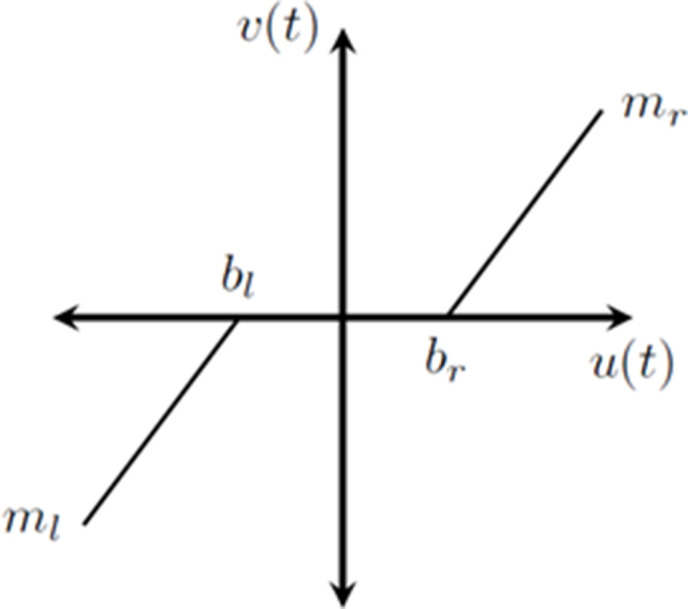
Smooth Dead Zone region of motors’ characteristic curve.

**Fig 3:**
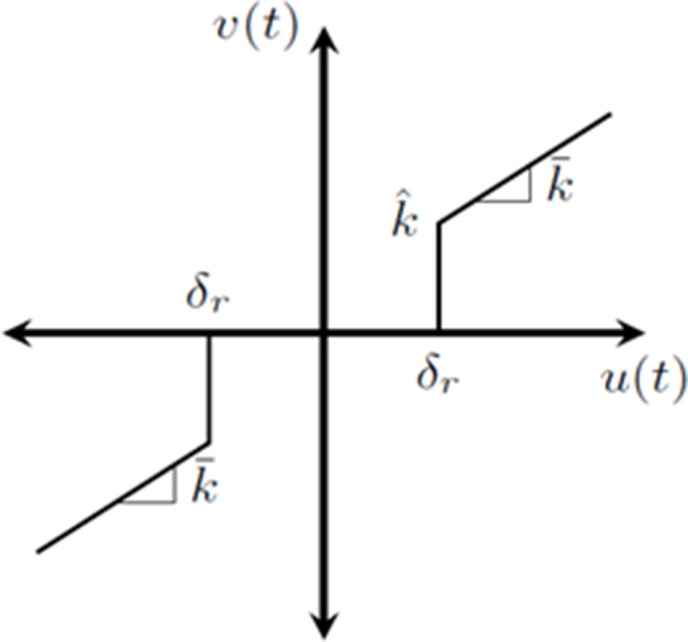
Hard Dead zone of PMDC motor characteristics.


The “smooth” dead zone depicted in Fig 2 represents the non-linear dead zone behavior of the PMDC motor where the input and output are denoted by u(t) and v(t), respectively, and the left and right break points are $b_{l}$ and $b_{r}$, and the dead zone’s slopes are $m_{l}$ and $m_{r}$. $m_{l}$= $m_{r}$ and $b_{l}\;=\;b_{r}$ when the dead zone is symmetric [18,19]. When it comes to electric motors, however, this approximation may not always correctly reflect the actual physical reality. Because of this, the dead zone is represented as the “hard dead zone” in Fig 3 [21], which is thought to be a accurate portrayal of the non-linear phenomenon that happened in the PMDC motor of all. In (7), the symmetric “hard dead zone” is provided.

$$v(t)=\left\{ \begin{array}{cc} \text{sin}\;n\left(u(t)\right)\left[\overset{―}{k}\left|u(t)\right|+\hat{k}\right] & \;\;\;\;\;;\;\left|u(t)\right|\geq \delta_{r}\\ \text{0} & \;\;\;\;;\;\left|u(t)\right|<\delta_{r} \end{array}\right.\;$$ (7)

where $\hat{k}$ denotes the system’s abrupt offset caused by breaking inertias, $\overset{―}{k}$ is the dead zone’s slope, δr denotes the dead zone’s break point, and u(t) is the system’s input and v(t) is its output.

#### 2.1.2. Friction modeling of PMDC motor.

**Fig 4:**
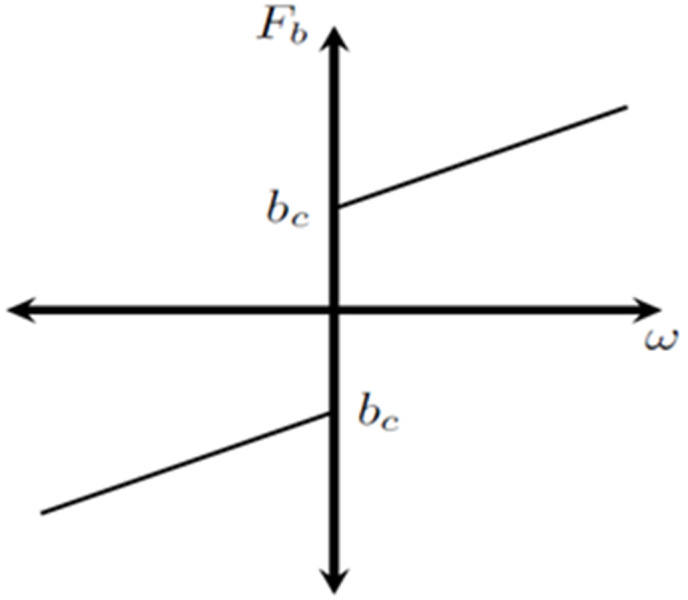
Characteristic curve for Coulomb and viscous friction.


The most widely used model of friction is the Coulomb plus viscous friction model, as illustrated in Fig 4, as friction is defined as the tangential reaction force between two surfaces in contact [22–24]. (8) gives a description of this model.

$$F_{b}=\left\{ \begin{array}{ccc} & b_{c}.\text{ sin}\;n(\omega )+b_{v}\;\omega & ;\;\omega \neq \text{0}\\ F_{ap} & & ;\;\omega =\text{0}\;and{\;F}_{ap}<b_{c} \end{array}\right.\;$$ (8)

where ω is the speed, $b_{v}$ is the viscous coefficient, $F_{ap}$ is the applied force, and $F_{b},\;b_{c}$, and $b_{v}$ are the friction forces and Coulomb friction forces, respectively [25–29].

#### 2.1.3. Linearization of PMDC motor model.

As the second validation stage of extended Kalman filter uses the linearized model of a nonlinear plant, therefore linearization framework and its implementation on PMDC motor is performed in this subsection. The generic nonlinear model is represented as in (9).


$$x_{t}=f\left(x_{t-\text{1}},u_{t-\text{1}},w_{t-\text{1}}\right)$$

$$y_{t}=h(x_{t},\;v_{t})$$ (9)

and corresponding linearized state space form is represented by (10).


$$\dot{x_{t}}=Ax_{t}+B{u_{t}+w}_{t}$$

$$\;y_{t}=Hx_{t}+v_{t}\;$$ (10)

where x is the system state matrix of order n x 1, F is the state coefficient matrix of order n x n, $x_{t}$ is the previous state matrix of order n x 1, B is the input coefficients matrix of order $n\;x\;m,\;u_{t}$ is the input matrix of order $m\;x\;\text{1},{\;w}_{t}$ is the process noise matrix of order $n\;x\;\text{1},\;z_{t}$ is the outputs matrix of order m x 1, H  is the output coefficient matrix of order $m\;x\;n,\;v_{t}$ is the output noise matrix of order m x 1. Where m denote the number of inputs and n denote the order of the system. The linearized form (10) is obtained from (9) by computing Jacobeans of the state(s)-input(s) map $f\left(x_{t-\text{1}},u_{t-\text{1}},w_{t-\text{1}}\right)$ and state(s)-output(s) map $h(x_{t},\;v_{t})$ as below:


A[i,j]=∂f[i](xt−1^,  ut−1,0)∂x[j], B[i,j]=∂f(xt−1^,ut−1,0)∂u[j], H[i,j]=∂h[i](xt^, 0)∂x[j]

These matrices are evaluated at Linearization point ($x_{t},u_{t}$)=(0,0) to get the linear form of nonlinear model. The linearized model of PMDC motor becomes as follows:


$$[\begin{array}{c} \dot{x_{\text{1}}}\\ \dot{x_{\text{2}}}\\ \dot{x_{\text{3}}} \end{array}]=[\begin{array}{ccc} \text{0} & \text{1} & \text{0}\\ \text{0} & -\frac{b}{J} & \frac{K_{T}}{J}\\ \text{0} & -\frac{K_{e}}{L} & -\frac{R}{L} \end{array}][\begin{array}{c} x_{\text{1}}\\ x_{\text{2}}\\ x_{\text{3}} \end{array}]+[\begin{array}{c} \text{0}\\ \text{0}\\ \frac{\text{1}}{L} \end{array}]u+[\begin{array}{c} \text{0}\\ -\frac{\text{1}}{J}\\ \text{0} \end{array}]d$$

$$y=[\begin{array}{ccc} \text{0} & \text{1} & \text{1} \end{array}][\begin{array}{c} x_{\text{1}}\\ x_{\text{2}}\\ x_{\text{3}} \end{array}]$$ (11)

During simulatory and experimental validation step, especially for velocity control scenario, it was observed that the open loop system given in (9) and (11) didn’t follow the reference input. Therefore, in order to make PMDC motor model follow the desired reference, PID controller is designed using the optimal auto-tuning method. The framework of this linear PID controller is discussed next.

### 2.2. Linear control of PMDC motor model

**Fig 5:**
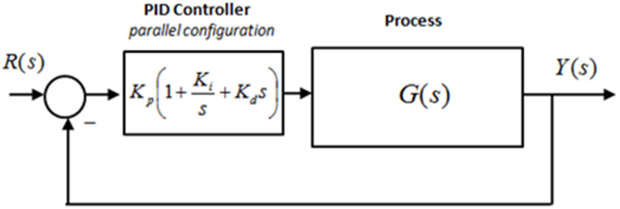
Structure for deployment of PID controller.

In order to achieve model output that follow the reference input, a PID controller is designed. PID controller produces a closed loop PMDC motor system that obeys its reference input or inputs. In contrast to two mode controllers like PI and PD Controller, the primary purpose of a PID controller is to offer reliable performance for all operating situations and complex processes in order to precisely produce the intended output. As seen in Fig 5 below, a PID controller determines a “error” value as the difference between a measured process variable (PV) and a desired set point.

With the integral action on error and the derivative action on error with regard to the process’s fixed point, the PID controller removes steady state error in any process. Equation (12) relates the error input e(t) and output y(t) in the PID Controller algorithm.


$$y(t)=K_{p}e(t)+K_{i\;}e\int e(t)\text{dt}+K_{d}\frac{\left(\text{de}(\text{t})\right)}{dt}$$ (12)

where, $K_{i\;}=\frac{\text{1}}{T_{i\;}}\;and\;K_{d\;}=T_{d}$ and in transfer function form the PID controller equation is given by:


$$G_{PID}=K_{p\;}+\frac{K_{i\;}}{\text{s}}+K_{d}s=\frac{K_{d}s^{\text{2}}+K_{p}s+K_{i}}{\text{s}}$$

There are several methods available for tuning the three mode controller parameters (Proportional gain, Integral gain, Derivative gain) and we utilize the auto-tuner block of Matlab to optimally tune the PID parameters in our work.

## 3. Improved noise reduction using variants of extended Kalman filter

This section provides the framework for reduction of measurement noise using two variants of EKF. Standard EKF method is described first and thereafter problems of noise estimation using this filter are highlighted. In last part of the section, an improved adaptive EKF version that better approximate noise statistics and provide relatively accurate estimation results is presented.

### 3.1. Development of standard extended Kalman filter algorithm

The role of EKF is to produce the best possible state/output estimate from noise corrupted outputs and inputs data. For estimation extended Kalman filter requires nonlinear/linearized system model, noise corrupted measurements, noise covariance of state’s error and outputs. Once these are known, Kalman filter recursively average the output corresponding to required unknown state. Process noise covariance (Q) and measurement noise covariance (R) are set based on user experience/plant’s dynamical behaviour and are mathematically stated by $Q=E[w_{t}w_{t}^{T}$] and $R=E[v_{t}v_{t}^{T}]$ respectively. As EKF is a recursive algorithm, we need to initialize state and error covariance matrix. The state error covariance is given by $P_{t}=E\left[e_{t}e_{t}^{T}\right]$, where $P_{t}$ is the error covariance, et=xt−xt^ is the difference between actual and estimated states, $x_{t}$ represent actual state matrix and xt^ represent estimated states matrix. The complete EKF algorithm for easy understanding is divided in two stages as discussed below:

1) Prior Estimation (Prediction Stage): After ${\hat{x}}_{t-\text{1}}$ and $P_{t-\text{1}}$ is initialized, Kalman filter compute xt^−(i.e., state estimate prior to the occurrence of output) and $P_{t}^{-}$(i.e., error covariance prior to the occurrence of output) using relations:


$$x_{t}=f\left(x_{t-\text{1}},u_{t-\text{1}},w_{t-\text{1}}\right)$$

$$P_{t}^{-}=AP_{t-\text{1}}A^{T}+Q$$

As this computation is performed before actual measurement time has arrived therefore we call this stage as “Prediction stage”.

2) Correction Stage: Now as actual output ($z_{t}$) arrives, the xt^− and $P_{t}^{-}$ are validated with available measured output, necessary scaling of error is done by multiplying ‘$K_{t}$’ (Kalman Gain) and is added with reference offset xt^− as below:


xt ^=xt ^ −+Kt(yt−Hxt ^ −)

In above relation, $K_{t}$ must be known to finalize the estimate, which is derived in four steps, i.e., Put xt^ in (i) and then put $e_{t}$ in $P_{t}=E[e_{t}e_{t}^{T}]$ and solve for${\;P}_{t}$. Now choose $K_{t}$ so that terms containing $K_{t}$ are zero and finally solve for $K_{t}$ to finally arrive at:


$$K_{t}=P_{t}^{-}H^{T}{\left(HP_{t}^{-}H^{T}+R\right)}^{-\text{1}}$$

Once $K_{t}$ is computed, putting it in (i) gives the required state estimate of immeasurable state.

The state error covariance matrix is also updated in correction stage by using relation:


$$P_{t}=\left(I-K_{t}H\right)P_{t}^{-}$$

Finally, state vector and error covariance index is updated for next iteration.

**Remark:** One drawback of using standard version of EKF is that it ignores the effect of noise in the physical system and setting process and measurement covariance values in a vague manner cause inaccurate estimates. In order to remedy this problem, an adaptive variant of EKF is introduced in next section that produce accurate estimation results.

One reason why adaptive EKF was used is that unlike the conventional EKF, which makes the assumption of constant process and measurement noise covariances, AEKF adapts these covariances in real-time, achieving accurate results in non-linear and time-varying systems when the characteristics of noise are unknown or vary with time. In comparison to UKF, which employs a sigma-point method for accuracy in highly non-linear systems, AEKF maintains computational efficiency while achieving better robustness under modeling uncertainties. As such, while Particle Filters [30–32] have better performance in highly non-Gaussian and multi-modal cases, they are too costly computationally and need many particles for accurate estimation. AEKF brings a realistic compromise by retaining the efficiency of the Kalman filter structure but offering considerable improvement in terms of adaptability, thus making it suited for real-world applications such as autonomous navigation, robots, sensor fusion, and aerospace systems where the statistics of the noise are not always known or change consistently. Its self-tuning capability improves robustness to environmental variations and sensor errors, providing more stable and consistent performance than other filters that are either non-adaptive or require significant computational power.

### 3.2. Development of adaptive extended Kalman filter algorithm

The adaptive extended Kalman filtering algorithm [33] estimates and corrects the process and measurement noise of the system by comparing the final estimates with the predictions. This reduces the error caused by the extended Kalman filtering algorithm ignoring the influence of noise in the actual process [34,35]. The significant enhancement comes from incorporating a forgetting factor. The forgetting factor in an adaptive Extended Kalman Filter (EKF) has a vital impact on noise cancellation by adaptively controlling the sensitivity of the filter to new data while suppressing the effect of older data. The process and measurement noise covariances in adaptive EKF are usually updated online to address non-stationarity in the noise statistics. The forgetting factor, usually a number between 0 and 1, determines how rapidly previous information is “forgotten” to make room for newer observations. A low forgetting factor assigns more importance to new measurements, making the filter very sensitive to abrupt changes in noise characteristics but at the cost of instability. Alternatively, a larger forgetting factor provides smoother updates by keeping the past data, preventing overfitting to temporal variations in noise. Such a balance is needed especially in dynamic scenarios, e.g., speech enhancement, biomedical signal processing, and radar tracking, where the characteristics of noise vary with time. By adjusting the forgetting factor appropriately, the adaptive EKF is able to suppress the noise while preserving strong state estimation and thus produce better real-world applications.

Aside from forgetting factor, other important parameters like process noise covariance Q and measurement noise covariance R have a direct impact on the filter’s capability to distinguish between system dynamics and noise. These parameters need to be well-tuned to ensure that the EKF is able to respond to changing levels of noise without losing estimation accuracy. If Q is too big, the filter can be too sensitive to noise and result in unstable estimates, while a small Q will make the filter insensitive to major changes in the system. Also, an unreasonably high R value will make the EKF over-dependent on the model and less responsive to actual observations, while a very low R will make it too sensitive to noisy measurements. Adaptive EKFs adapt these parameters dynamically with methods like innovation-based adaptation, maximum likelihood estimation, or machine learning-based approaches [36–38], thus enhancing noise robustness and improving filtering performance in real-time. Ideally tuned parameters guarantee that the EKF is stable in a wide range of different noise environments, resulting in optimal signal reconstruction and better noise cancellation.

In order to minimize the effect of noise on state-of-charge estimation and increase the accuracy of the state-of-charge estimation results, process noise and measurement noise of the system are evaluated and corrected at the same time. Average estimation and covariance are also modified. This work use weighting coefficients [39,40] to lessen the weight of noise at time k in order to more properly represent the influence of noise due to an inadvertent measurement error. The calculation formula for this is provided in Eq. (13).


$$d_{t-\text{1}}=\frac{\text{1}-b}{\text{1}-b^{t}}$$ (13)

where the element of forgetting is located in b. The influence of the preceding moment is less in real-world applications the lower the value of b. The anticipated noise will, however, change with a tiny value of b. The preceding moment’s impact will be excessive if b has an excessively high value. The value can therefore be determined based on the particular circumstances. Equation (14), following the necessary adjustments, displays the noise matrix approximation/computation formula.


$$\begin{array}{c} q_{t}=\left(\text{1}-d_{t-\text{1}}\right)q_{t-\text{1}}+d_{t-\text{1}}G({\hat{x}}_{t}-A{\hat{x}}_{t-\text{1}}-Bu_{t-\text{1}}) \end{array}$$

$$\begin{array}{c} Q_{t}=\left(\text{1}-d_{t-\text{1}}\right)Q_{t-\text{1}}+d_{t-\text{1}}G\left(K_{t}y_{t}y_{t}^{T}K_{t}^{T}+{\tilde{P\;}}_{t-\text{1}}-A{\tilde{P\;}}_{t-\text{1}}A^{T}\;\right)G^{T} \end{array}$$

$$\begin{array}{c} r_{t}=\left(\text{1}-d_{t-\text{1}}\right)r_{t-\text{1}}+d_{t-\text{1}}(y_{t}-C{\hat{x}}_{t}-Du_{t}) \end{array}$$

$$\begin{array}{c} R_{t}=\left(\text{1}-d_{t-\text{1}}\right)R_{t-\text{1}}+d_{t-\text{1}}(y_{t}y_{t}^{T}-C{\tilde{P\;}}_{t-\text{1}}C^{T}) \end{array}$$ (14)

where $q_{t-\text{1}}\;$is the system state noise, ${\hat{x}}_{t}$ is the state of the system at timet, A is the system state transition matrix, and B is the control matrix. $Q_{t}$ is the covariance matrix of system state noise, $y_{t}$ is the state observation measurement, and G is the noise driven matrix. $P_{t-\text{1}}$ is the error covariance matrix of initial prediction. $r_{t}$ is the system observation noise, and H is the system measurement matrix. $R_{t}$ is the covariance matrix of system observation noise. Once the noise correction is applied, rest of the estimation process is quiet similar to standard EKF consisting of prediction and correction stages as re-iterated as described in section 3.1. However, at final step in adaptive EKF, apart from updating state and state error covariance values, in adaptive EKF values of $q_{t},\;r_{t},\;Q_{t}$ and $R_{t}$ matrices are also updated as given in Eq. (14).

It should be noted that adaptive EKF functions under some essential assumptions in order to properly tune noise covariance matrices and improve state estimation precision. It initially considers that the system takes a non-linear state-space model like the conventional Extended Kalman Filter (EKF), with process and measurement noises being represented by Gaussian distributions. But in contrast to traditional EKF, AEKF postulates that such noise covariances are not stationary and may vary over time. It also banks on the premise that statistical methods based on innovation, e.g., maximum likelihood estimation or covariance matching, can yield useful updates for such noise parameters. AEKF further assumes that real-time measurements are rich in information that will enable estimation and adaptation of the noise properties without causing instability. Though these assumptions render AEKF extremely flexible and robust, its performance may be compromised if noise distributions vastly depart from Gaussianity or if system dynamics are not sufficiently observable to accurately tune covariances.

Forgetting factor adjustment within an adaptive EKF is essential to achieve responsiveness and stability. A lower forgetting factor (nearer 0) enables the filter to quickly respond to changes in system dynamics or noise patterns but can cause instability or amplify transient noise. A higher forgetting factor (nearer 1) prefers stability and smoothness but can make the filter slow in responding to abrupt changes. It is advisable to start with a moderate value (say, 0.95–0.99) and then vary according to system behavior through empirical testing or through performance metrics such as RMSE. Further, application of a variable forgetting factor strategy—where the factor varies according to residuals or error in estimation—can improve performance in dynamic regimes as well.

Regarding the practical guidelines for setting parameter values for an adaptive EKF, first consideration requires a balance of accuracy, stability, and adaptability to varying noise conditions. A practical approach begins with an initial estimation of process noise covariance Q and measurement noise covariance R, which can be derived from system characteristics, empirical data, or domain expertise. Typically, Q should reflect the level of uncertainty in the system dynamics—higher values make the filter more responsive to changes but may introduce instability, whereas lower values result in smoother estimates at the risk of lagging behind real changes. Similarly, RR should correspond to sensor noise characteristics, where an overly high R value will cause the filter to rely too much on the model, while a very low value can lead to overfitting to noisy observations. To adapt these parameters dynamically, innovation-based methods (e.g., covariance matching) or machine learning techniques can be employed, ensuring real-time adjustments based on observed system behavior. Regular performance evaluation using metrics like root mean square error (RMSE) and residual analysis can guide further fine-tuning. Additionally, practical implementations often benefit from a bounded adaptation approach, where Q and R are allowed to vary within predefined limits to prevent instability. Testing under different noise conditions and applying domain-specific knowledge are essential for achieving robust and reliable filtering performance.

The input-output dataset of the PMDC motor inclusion criteria in noise estimation with an adaptive Extended Kalman Filter (EKF) aim at recording representative behavior of the motor under different operating conditions. Such datasets involve varying load torques, input voltage, and speeds to provide strong resistance towards noise modeling. Signals should be sampled at a high enough frequency to maintain system dynamics and both voltage and current as inputs and angular velocity or position as output. Exclusion criteria include the removal of data that have missing values, sensor failures, or anomalous transients not typical of normal operation since they will bias the EKF’s adaptability to noise and diminish filter performance.

## 4. Results and discussions

**Fig 6:**
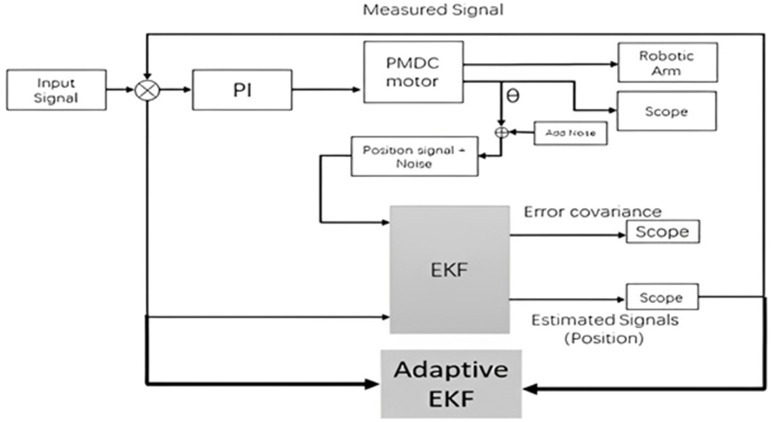
Procedure for simulation and experimentation.

The propositions of above sections have been tested through simulations as well as through experimental setup. The sequence of simulatory and experimental setup is shown in Fig 6.

**Fig 7:**
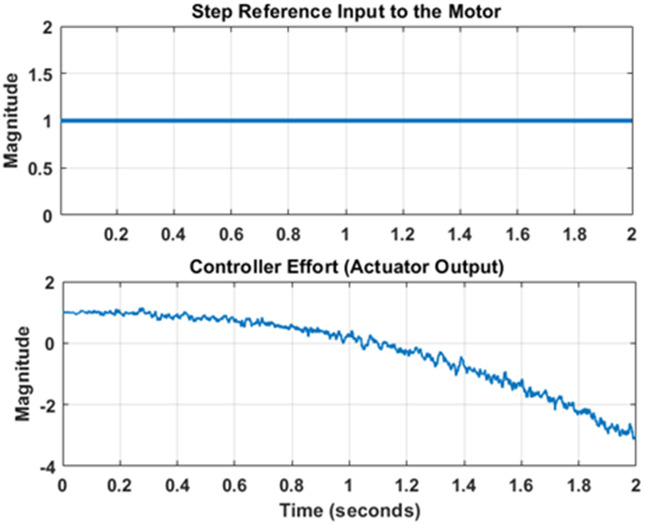
Reference input and Actuator Output.

**Fig 8:**
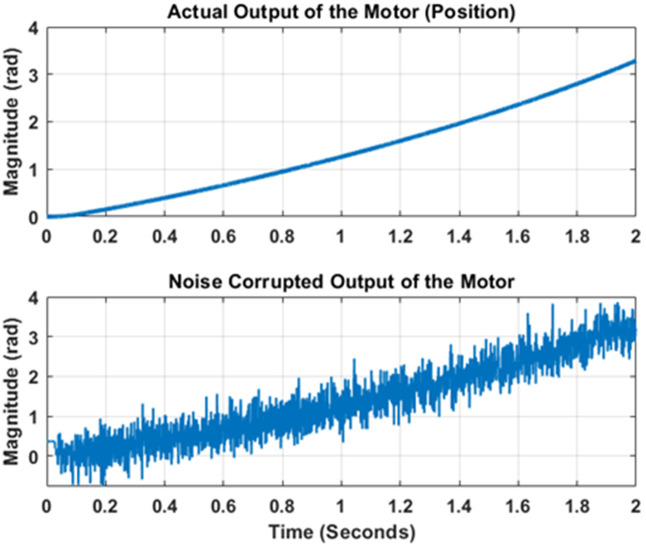
Actual and Noisy angular position outputs.

***Regarding the simulatory setup***, the nonlinear model of PMDC motor presented in section 2 is supplied with a step input representing a constant DC voltage. The PID controller parameters are tuned by using the Ziegler Nicholas method. The reference DC input and controller effort signals have been shown in Fig 7. Out of the four possible outputs, the angular position output of the model is selected. This output gets contaminated after interaction with process and measurement noise. The true and noisy outputs have been shown in Fig 8. Note in Fig 8 that the angular position increases as DC input has been continuously applied. The state and output error covariance has been taken as $Q=[\begin{array}{cc} {\text{1}\text{0}}^{-\text{6}} & \text{0}\\ \text{0} & {\text{1}\text{0}}^{-\text{2}} \end{array}],\;R={\text{1}\text{0}}^{-\text{4}}$.

**Fig 9:**
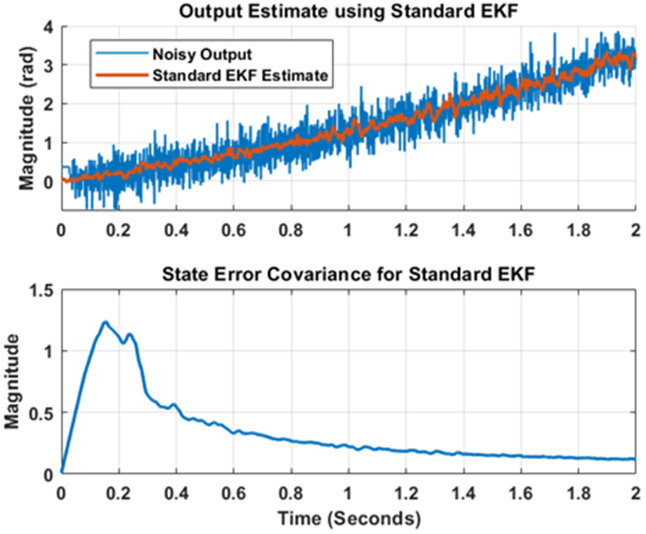
Output estimation using Standard EKF.

The standard EKF algorithm is applied to the model by invoking input and noise corrupted output data to it. The covariance’s are again set as detailed above and the estimate of the angular position obtained as filtered signals generated from standard EKF version are depicted in upper portion of Fig 9. The red line shows that the EKF is very comprehensively cancelling the noise in measurement and directing user to the true value. The lower portion of Fig 9 shows the state error covariance for standard EKF estimator. The graph shows that the error covariance is decreasing as time progresses, which validate the successful implementation of the filter as state estimator.

**Fig 10:**
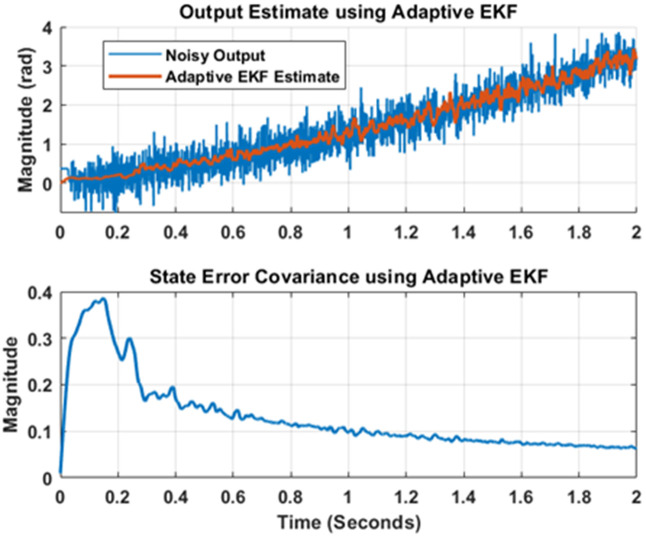
Output estimation using Adaptive EKF.

Furthermore, the novel adaptive EKF algorithm that takes care of noise estimation is applied to the model by invoking plant input and noise corrupted output. The forgetting factor tuning is performed to obtain the closest possible output estimate to the true reading. The results after tuning are being shown in Fig 10. The upper portion of Fig 10 shows that the estimator results (in red) are promising and estimator is comprehensively rejecting the measurement noise. The state error covariance plot for the adaptive EKF implementation is shown in lower part of Fig 10. The covariance decay as time progresses that again validate the successful implementation of the adaptive EKF algorithm.

**Fig 11:**
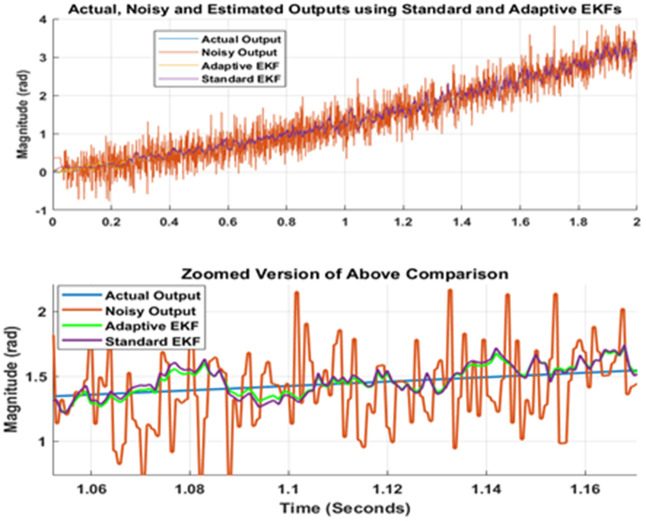
Comparison of estimation results for standard and adaptive EKFs.

**Fig 12:**
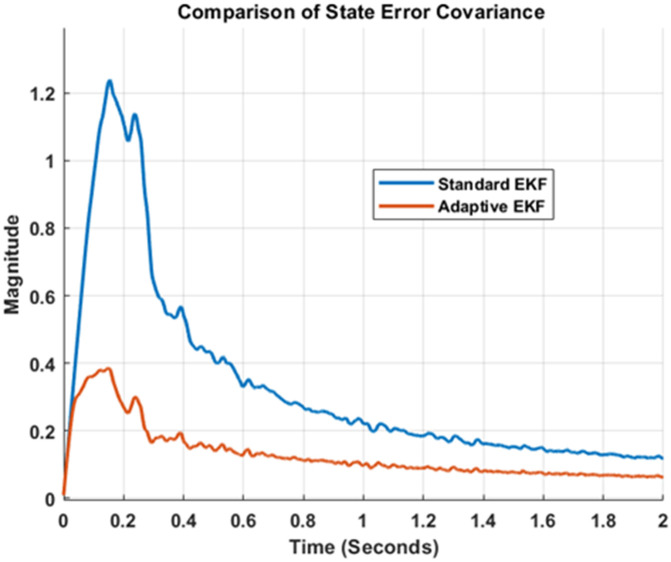
Comparison of state error covariance for standard and adaptive EKFs.

Finally, the comparison of standard and adaptive EKF performances has been shown in Figs 11 and 12. Upper part of Fig 11 shows the actual, noisy and estimated outputs generated by standard and adaptive EKFs. To clarify the graph, lower portion of Fig 11 present zoomed version of the responses. Note that the adaptive EKF produce better position estimates that are more close to true value as compared to standard EKF. Furthermore, Fig 12 depicts the comparison of state error covariance’s for both estimators. This Fig once again validates the successful implementation of both filters as well as superiority of adaptive filter over traditional standard version of estimator.

**Fig 13:**
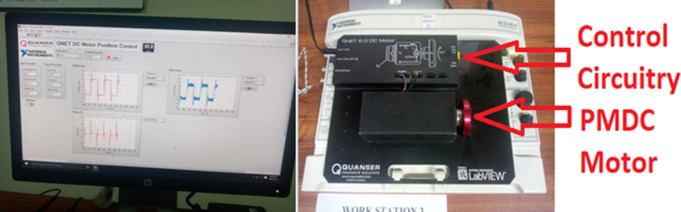
Experimental Setup for noise reduction in position and velocity scenarios. Note in third part of Fig 14 that an overshoot is noted due to inertia when motor reach its reference angle when it is reversed. Also the controller output/effort change its polarity as motor reverses its direction. Furthermore, when noisy output and reference input is applied to the nonlinear variants of Kalman estimators, estimation results from standard and adaptive EKF are obtained that are shown in Fig 15. Fig 15 shows that the proposed adaptive EKF more comprehensively estimate the true reference value as well as provide less overshoot at reversal points. Thus the relatively better performance of proposed EKF variant is validated. The state.

**Fig 14:**
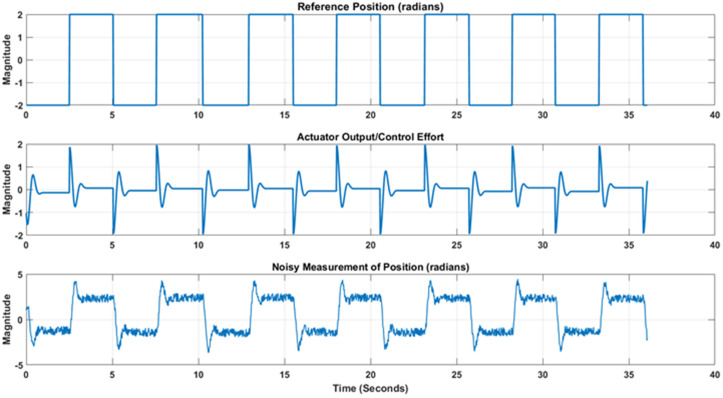
Reference input, controller effort and noise corrupted measured position signals.

***The experimental results*** for position as well as velocity control for Quanser QNET DC motor activated and interfaced to LabVIEW through NI Elvis II kit are validated for standard and adaptive extended Kalman filter versions. The experimental setup has been shown in Fig 13. Auto-tuning for optimized performance of PID controller for both position and velocity scenarios is performed. Firstly, position control and estimation scenario is presented. The driving reference position signal with switching polarity square wave is applied to the motor through the tuned PID closed loop in order to rigorously assess the validity of our designed controller and estimator. The noise corrupted position output signal was acquired in LabVIEW and the reference signal, respective controller effort and noise corrupted measured position signals have been shown in Fig 14.

**Fig 15:**
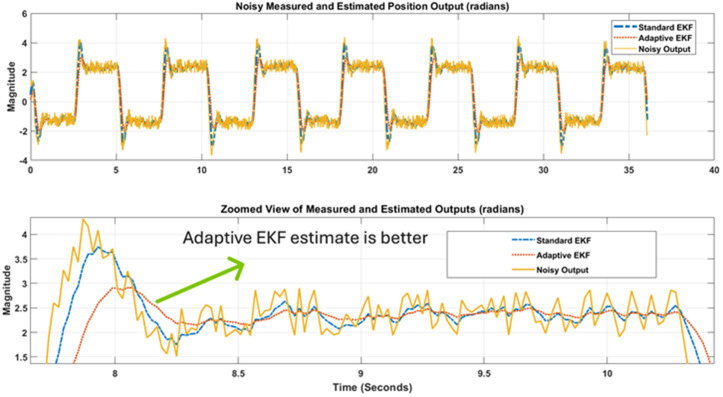
Noise reduction performance of Standard and Adaptive EKFs for position output.

error covariance plots for standard and adaptive EKFs have been depicted in Fig 16 that again confirms that the error covariance of adaptive EKF is less that the standard version.

**Fig 16:**
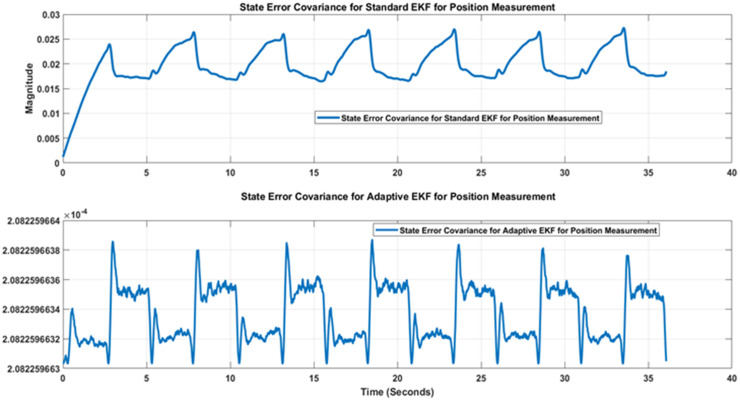
Comparison of State error covariance of Standard and Adaptive EKFs for position.

**Fig 17:**
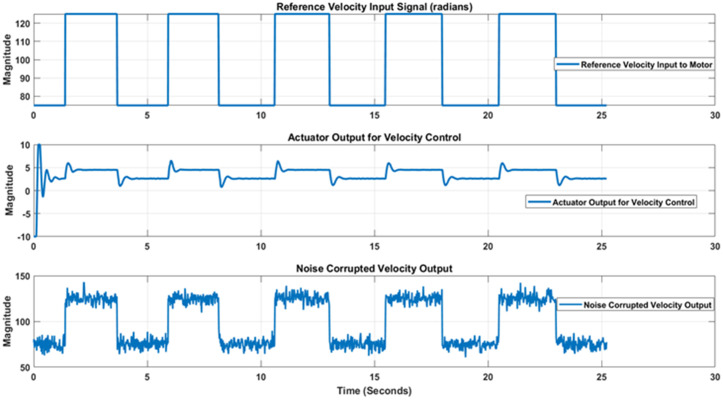
Reference input, controller effort and noise corrupted measured Velocity signals.

For velocity measurement, control and estimation case the motor was driven with the reference velocity switching from 75 rads/sec to 125 rads/sec forming a square waveform. The PID controller was auto-tuned and noise corrupted velocity signal was measured at the output. The reference signal, respective controller effort and noise corrupted measured position signals have been shown in Fig 17.

**Fig 18:**
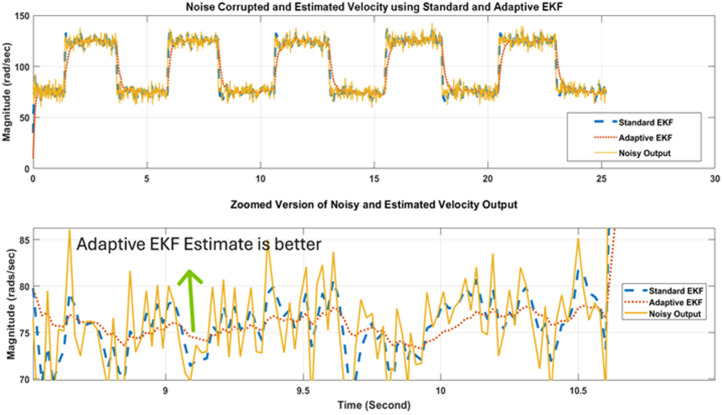
Performance comparison of Standard and Adaptive EKFs for velocity output.

**Fig 19:**
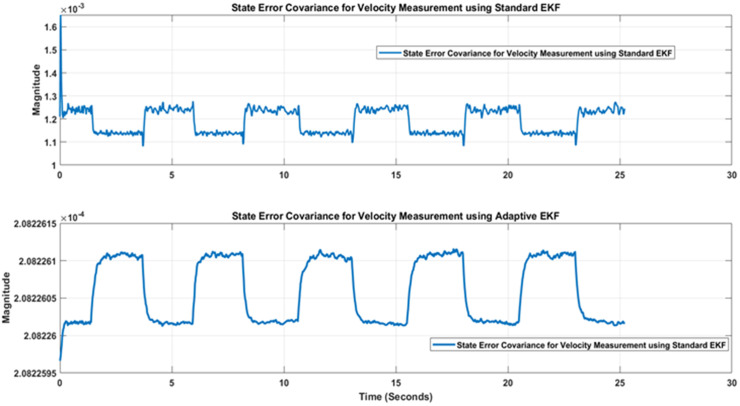
Comparison of State error covariance of Standard and Adaptive EKFs for velocity.

In a similar way to position measurement scenario, when noisy output and reference input is applied to the standard and adaptive variants of Kalman filter, respective estimation results are obtained that are shown in Fig 18. Fig 18 again shows that the proposed adaptive EKF more comprehensively estimate the true reference velocity value in both directions as well as provide less overshoot at reversal points. Thus the relatively better performance of proposed EKF variant is validated for velocity scenario as well. Furthermore, the state error covariance plots for velocity measurement scenario for standard and adaptive EKFs have been depicted in Fig 19 that again confirms that the error covariance of adaptive EKF is less that the standard version.

Results presented in this section confirm that the adaptive AEKF provides substantial quantitative enhancements to the traditional EKF by adaptively adjusting noise covariance matrices, resulting in improved estimation accuracy and robustness. In contrast to the traditional EKF, which uses fixed process and measurement noise covariances, AEKF adapts these parameters in real-time using system dynamics and innovation statistics. This adaptation minimizes estimation errors, especially in non-linear and time-varying systems, where model uncertainties and measurement noise vary. Research has demonstrated that AEKF can provide lower root mean square errors in state estimation, faster convergence speed, and higher robustness to unforeseen disturbances. These advantages make AEKF especially useful in applications like navigation, robotics, and sensor fusion, where accurate and reliable state estimation is essential. The table presenting the statistical analysis regarding comparison of Kalman filter, EKF and AEKF variants deployed for noise estimation of PMDC motor is given below Table 1:

**Table 1:** Statistical performance analysis KF variants for noise estimation and rejection.

Metric	KF	EKF	Adaptive EKF	Remarks
Mean Absolute Error	0.072 rad/s	0.059 rad/s	0.038 rad/s	Adaptive EKF can better handle time-varying noise and nonlinearities in the model.
Root Mean Square Error	0.093 rad/s	0.076 rad/s	0.045 rad/s	The improvement in RMSE shows better precision in the estimation of dynamic states.
Steady-State Bias	0.018 rad/s	0.012 rad/s	0.005 rad/s	Lower bias means closer tracking of the real motor states.
Standard Deviation of Error	0.031	0.024	0.015	It depicts narrower spread of error with the adaptive EKF.
Convergence Time (ms)	220	180	130	Adaptive EKF very quickly adapts to system dynamics.
Noise Covariance Adaptation	Fixed	Fixed	Online adaptive (Q, R)	The adaptive EKF is the only one that learns and updates noise models in real-time.
Model Complexity	Low	Medium	High	Adaptive EKF needs more computation time (due to Q/R estimation logic).
Robustness to Noise Variance	Poor (Fixed Q/R)	Moderate	High (Dynamic Q/R tuning)	Adaptive EKF handles time-varying or uncertain noise better.
Computational Time per Step	0.52 ms	0.73 ms	1.25 ms	Additional time to update noise models is paid, but it’s within real-time control constraints.
Suitability for Nonlinear Systems	Poor	Good	Excellent	Kalman filter linearizes; adaptive EKF adapts both linearization and noise covariances.

## 5. Conclusions and future directions

This paper presents an improved framework for measurement noise reduction of nonlinear PMDC motor using standard and adaptive variants of extended Kalman filter (EKF). One of the contributions presented is the consideration of nonlinearities like hard dead zone and friction in the PMDC motor model. The noise estimation process becomes complicated for these nonlinearities, therefore adaptive EKF variant with best possible choice of forgetting factor has been invoked theoretically and experimentally. The performance superiority of proposed tuning structure for noise cancellation adds another contribution to the literature. Position as well as velocity measurement scenarios have been considered. At first, the noise corrupted measurement is invoked in standard EKF that perform prediction and correction to generate the best possible reduced noise estimate of the true measurement. One drawback standard EKF is that it ignores the effect of noise in the physical system and setting process and measurement covariance values in a vague manner that cause inaccurate estimates. In order to remedy this problem, an adaptive variant of EKF is introduced that produce relatively accurate results. The propositions are tested for angular position and velocity applications through simulation as well as practical experimentation. The presented developments of AEKF in this paper are valuable in applications of PMDC machines, navigation systems, robotics, and sensor fusion, where precise and reliable state estimation is critical. Conversely, future work on Adaptive EKF can be directed towards enhancing its adaptability to strongly non-Gaussian noise, increasing computational efficiency for real-time processing, and incorporating machine learning methods for dynamic noise estimation. Hybrid methods that combine AEKF with other filtering algorithms, like Unscented Kalman Filter or Particle Filter, can also be explored to further improve robustness. Moreover, a systematic method to tune the forgetting factor need also be devised in the future so that optimal noise cancellation can be achieved.
